# The Influence of Omega-3 Long-Chain Polyunsaturated Fatty Acid, Docosahexaenoic Acid, on Child Behavioral Functioning: A Review of Randomized Controlled Trials of DHA Supplementation in Pregnancy, the Neonatal Period and Infancy

**DOI:** 10.3390/nu13020415

**Published:** 2021-01-28

**Authors:** Jacqueline F. Gould, Rachel M. Roberts, Maria Makrides

**Affiliations:** 1 Women and Kids, South Australian Health and Medical Research Institute, 72 King William Road, 5006 Adelaide, Australia; maria.makrides@sahmri.com; 2 School of Psychology and Discipline of Paediatrics, Faculty of Health and Medical Sciences, The University of Adelaide, 5005 Adelaide, Australia; 3 School of Psychology, Faculty of Health and Medical Sciences, The University of Adelaide, 5005 Adelaide, Australia; Rachel.roberts@adelaide.edu.au; 4 Discipline of Paediatrics, Faculty of Health and Medical Sciences, The University of Adelaide, 5005 Adelaide, Australia

**Keywords:** DHA, omega-3 fatty acids, supplementation, behavior, behavioral problems, prenatal, postnatal, neonatal, infant

## Abstract

This is a review of randomized controlled trials using docosahexaenoic acid (DHA) interventions in the first 1000 days of life with assessments of behavioral functioning in childhood. Electronic databases were searched for trials with a DHA intervention (compared with a placebo group that received no or less DHA) at any time to either women or infants during the first 1000 days, with a subsequent assessment of child behavior. There were 25 trials involving 10,320 mother–child pairs, and 71 assessments of behavior in 6867 of the children (66.5% of those originally enrolled). From the 71 assessments administered, there were 401 comparisons between a DHA group and a control group, with most reporting a null effect. There were no findings of a positive effect of DHA, and 23 instances where the DHA group had worse scores compared with the control group. There was limited evidence that DHA supplementation had any effect on behavioral development, although two of the largest trials with behavioral measures detected adverse effects. Future trials, and future follow-ups of existing trials, should make an effort to evaluate the effect of DHA intervention on behavioral functioning.

## 1. Introduction

Nutrition during the first 1000 days is considered to be the most influential (non-genetic) determinant of development [1,2,3,4,5,6]. Docosahexaenoic acid (DHA, 22:6n−3) is one nutrient that has been identified as crucial for neurodevelopment during this key phase [7]. DHA is accumulated in neural tissue across childhood, but is accreted most rapidly between the last trimester through to 24 months of age [8,9,10,11]. By the end of the first 1000 days, the brain has grown to 80% of its adult size [12] and although neurodevelopment continues across childhood, the foundations for later development are laid during this early period. Inadequate nutrition during the first 1000 days is linked with poorer developmental outcomes for children, even when nutritional status is subsequently corrected [4,13,14,15,16,17,18]. In the case of inadequate DHA and brain development, animal studies have demonstrated that severe prenatal deprivation results in reduced neural tissue DHA in the offspring [19,20,21,22,23]. Likewise, infants born preterm who are deprived of the placental supply of DHA during the fetal brain growth spurt subsequently have lower neural tissue DHA levels than term-born infants [9]. Children born preterm also have an increased risk of poor behavioral functioning and behavioral problems when compared with their term-born counterparts [24,25,26,27,28,29]. Behavioral problems can adversely impact school performance, peer relationships, mental health, and even employment and income in adulthood [30]. Management of behavioral problems typically involves multiple ongoing strategies targeting individual symptoms of a disorder involving the individual, their parents, and often their school. Such strategies are time and resource intensive, and hence there are often barriers to their access and implementation. It is important to evaluate potential protective factors to promote optimal behavioral functioning. Ensuring appropriate DHA during early brain development may be one strategy to contribute to optimal behavioral functioning [10].

### 1.1. Behavioral and Emotional Functioning

Behavioral and emotional functioning generally refers to an individual’s conduct and includes self-regulation of reactions to emotions and situations or environments, for example regulating frustration or concentrating on a task. An individual’s self-regulation and reactivity are often considered aspects of temperament as well as behavior. Behavioral functioning naturally improves with age across early childhood as an individual learns to regulate emotions, understands how to play with other children and builds relationships, and develops the ability to imagine the perspectives of others. Challenging behaviors such as having a tantrum or opposing parental instructions are common in childhood and are a part of normal development. When challenging behaviors are more frequent or severe, they can impact a child’s functioning and be diagnosed as a behavioral problem. The most commonly occurring behavioral problems to be diagnosed in childhood are Attention-Deficit/Hyperactivity Disorder (ADHD; inattention, which may or may not coincide with impulsive behaviors and hyperactivity), Autism Spectrum Disorders (ASD; a continuum of neurodevelopmental disorders with deficits in social engagement and communication as well as repetitive behaviors), Anxiety Disorders (overly fearful or worried), Oppositional Defiant Disorder (disobedience and resistance to authority) and Conduct Disorder (anti-social behaviors that violate rules or the rights of others). Behavioral problems are typically categorized into either internalizing (primarily affect the internal psychological environment, for example anxiousness) or externalizing problems (primarily outwardly expressed such as aggression).

### 1.2. The Link between DHA and Behavior

DHA is a structural component of the phospholipid bilayer of all cell membranes as it has an integral role in membrane fluidity [31]. Although present throughout the body, DHA is concentrated in the cells of the central nervous system, particularly the brain, where it is a prominent fatty acid [32,33]. DHA is essential for the growth and development of the CNS and brain [34,35,36]. DHA is known to be involved in neurogenesis, signal transduction and neurotransmission [37] and can enhance synaptic functions [38]; protect neural cells from apoptotic death [39], and oxidative stress [37]; induce synaptic growth cones during neuronal development [40,41]; influence neuron size [42,43]; stimulate neurite outgrowth in PC12 cells [44]; regulate nerve growth factor [45], membrane-bound enzymes [32], and ion channels [46] as well as dopaminergic and serotinergic neurotransmission [47,48].

The development of behavioral functioning is complex and likely involves the interplay of social, psychological and biological factors. Nutrients, such as DHA, may be one modifiable contributor to behavioral development. Observational studies have indicated a role for DHA in the pathology of numerous mental health issues [49,50,51,52,53]. Case–control studies have demonstrated higher DHA status in controls when compared with children with ASD [54] and ADHD [55] and it has been hypothesized that the rise in reports of ASD and ADHD may be in part due to the imbalance of omega-3 and omega-6 fatty acids in current Western diets [10,52]. See Cardoso et al. for a more in-depth review of observational associations between DHA and child behavior, as well as a summary of the possible biological mechanisms that may explain the role of DHA in behavioral disorders [10]. Interventional studies have explored DHA supplementation in childhood to assist in the treatment of behavioral disorders [10], with both benefits [56,57] and null [58,59] effects reported. As yet, there is little evidence to support the use of DHA supplements in the treatment of behavioral disorders such as ASD [58], learning disorders [59] and ADHD [57].

Whilst it is not uncommon for associations to be detected between behavioral problems and concurrent DHA levels or DHA intake, prospective longitudinal studies are needed to determine whether DHA exposure during the critical period of brain development can prevent or reduce the symptoms of behavioral functioning. Observational studies of prenatal DHA intake or status have assessed a range of developmental outcomes, but mainly focusing on cognitive abilities [16,17,60,61,62,63,64]. The largest and most robust observational study explored intake of fish and seafood (naturally rich sources of DHA) in 5499 pregnant women and found that low intake was associated with poorer behavior in children at 8 years of age [16]. A smaller observational study has similarly reported that increased prenatal dietary DHA intake is associated with reduced hyperactivity at 9 years [62]. Other observational studies of DHA status and the omega-3:omega-6 ratio fatty acid in cord blood have reported associations with reduced parent-rated symptoms of ADHD [65] as well as internalizing behavior problems [66] at 7 years of age. As many factors that are associated with dietary DHA in early life are also associated with behavioral outcomes (for example socioeconomic status), randomized controlled trials (RCTs) are needed to establish a causal pathway between early DHA exposure and later child behavioral development [67].

There have been many DHA interventions evaluated in the first 1000 days, but to date all have been restricted to a specific window, such as during pregnancy [68,69], with preterm-born neonates (<37 weeks’ completed gestation) [70], to breastfeeding women [71], and during infancy and/or toddlerhood [72]. Trials that have explored the effect of early DHA supplementation on subsequent brain development have been focused on cognitive assessments. Although occasional benefits have been reported, results are predominantly null and the consensus from reviews is that the evidence does not support the hypothesis that early DHA supplementation improves brain development [68,69,70,71,72,73,74]. The focus on cognitive outcomes as an indication on brain development is partly driven by the importance given to cognitive functioning for educational, general health and work outcomes, as well as economic benefits and societal contributions [75] and partly because the areas of the brain undergoing rapid development during the first 1000 days are the regions considered responsible for governing cognition. Hence nutritional deficiency during this time has been shown to have irreversible effects on cognitive outcomes [4]. However, the neurological areas of the brain responsible for behavior are similarly developing rapidly across the first 1000 days. Yet behavioral functioning has only occasionally been included as an exploratory outcome in some DHA intervention RCTs in early life.

There has been no consideration given to collating and considering the evidence for DHA supplementation in early life to effect development of child behavior as a whole [68,69,70,71,72,74,76], despite the substantial interest in the role of DHA for behavioral problems [10,52,53,57,58,59,77]. Furthermore, whilst adequate DHA is likely to be important throughout the whole of the first 1000 days, only two reviews to date have attempted to synthesize the evidence from RCTs conducted across this period and behavior was not a reported outcome of either review [76,78]. Given the biological plausibility for the role of DHA in behavioral functioning as well as the increasing body of evidence supporting the importance of this role, further research is warranted [10]. This review will be the first to amalgamate the totality of the evidence examining the effect of early DHA supplementation on behavioral functioning in childhood. We review RCTs with a DHA intervention during the first 1000 days that include a measure of any aspect of behavioral functioning, or behavioral problems.

## 2. Materials and Methods

Our review was undertaken according to the Preferred Reporting Items for Systematic Reviews and Meta-Analysis (PRISMA) guidelines [79].

### 2.1. Search Strategy

The Cochrane Central Register of Controlled Trials (CENTRAL) Current Contents Connect, PsychInfo, and PubMed were searched for eligible RCTs in humans that were written in English. Search results were screened to assess eligibility through the titles and abstracts. Additionally, given that behavior is rarely a primary or main outcome of DHA intervention studies, we reviewed the full text of any DHA RCTs that reported child developmental outcomes. Where only abstracts were accessible, such as from conference proceedings, these were included. Reference lists of included articles returned by the search were checked for other eligible articles as well as reference lists of other reviews [52,68,69,71,72,76,78]. PubMed was set up to email new publications identified by the search on a monthly basis with new articles added to the review up until acceptance of the manuscript.

### 2.2. Inclusion and Exclusion Criteria

Studies were eligible for inclusion in our review if an RCT in humans that was published in English, included supplementation with DHA (or long-chain polyunsaturated fatty acids in conjunction with DHA), included a placebo group without (or with less) DHA (or other fatty acids), and where the intervention occurred during any time within the first 1000 days to either a mother or infant (so long as target recipient of the intervention was the offspring), and the study reported a measure of child behavioral functioning, including temperament and emotional functioning.

### 2.3. Data Extraction and Synthesis

Study details (such as study characteristics, intervention details, results and possible sources of bias) were extracted from eligible studies into a table by one author (JFG). Included studies were categorized and synthesized according to the intervention period as (1) maternal interventions during pregnancy, (2) interventions for neonates born preterm, (3) postnatal interventions for breastfeeding mothers, and (4) postnatal interventions directly to infants or toddlers. Where interventions spanned more than one of these categories (for example throughout pregnancy and breastfeeding), the RCT was considered according to the period when the intervention commenced.

Assessments of behavioral functioning can be based on caregiver report, on teacher ratings, clinician administered or self-reported by the individual. Caregiver reports have the benefit of providing information about behavior in the child’s home environment but are a subjective measure. Teacher reports are ideal for gauging the severity of symptoms in a classroom environment and have the advantage that a teacher is likely comparing the individual’s behavior to their age-matched peers. Clinician reports are the least subjective but are least likely to reflect everyday behavioral functioning in an everyday environment. Behavior assessments can capture overall functioning, or a specific domain (such as only externalizing or only internalizing) or can target the specific symptoms of a specific behavioral disorder such as ADHD. Many assessments are age-normed (often sex-specific, indicating whether the behavioral functioning of an individual child is appropriate for a typical child of the same age). Unusual (extreme) scores can indicate a possible behavioral problem. However, clinical diagnosis involves a range of clinician-administered assessments and interviews to determine whether an individual meets the criteria to be classified according to the Diagnostic and Statistical Manual of Mental Disorders, now in its fifth edition. Outcome assessments of included studies were categorized and discussed as

(a) Diagnosed behavioral problems (such as by a psychologist, psychiatrist or pediatrician) or use of prescription medication for a behavioral problem,
(b) Clinician-administered general behavior measures and/or behavioral sub-scores of other measures,
(c) Teacher-rated behavior measures,
(d) Parent-rated behavior measures,
(e) Self-reported behavior measures, and
(f) Sensitivity and/or subgroup analyses.

## 3. Results

Our search identified a total of 25 RCTs as eligible for inclusion, with relevant trial outcomes or details published between 1999 and 2020 across 36 publications [80,81,82,83,84,85,86,87,88,89,90,91,92,93,94,95,96,97,98,99,100,101,102,103,104,105,106,107,108,109,110,111,112,113,114,115]. We excluded two potentially eligible trials, one that intervened with eggs (which contain DHA as well as other nutrients that contribute to neurodevelopment) vs. no control eggs [116] and another that commenced the intervention for the majority of children after the first 1000 days period (18–38 months, mean 27 months) [117].

### 3.1. Characteristics of Included Studies

The 25 RCTs (see Table 1) enrolled a total of 10,320 participants and were predominantly conducted in high-income countries (the United States (USA), Australia, Norway, Denmark, the United Kingdom (UK), Germany, and the Netherlands) whilst five were undertaken in low- or middle-income countries (Iran, Mexico, Bangladesh, Malawi, and Ethiopia) [80,89,90,93,106,115].

### 3.2. Sample and DHA Supplementation Details

#### 3.2.1. Maternal Interventions during Pregnancy

There were 10 RCTs investigating the effect of prenatal supplementation in 5913 pregnant women [80,81,82,83,84,85,86,87,88,89,90,91,92,93,94].

Pregnant women were asked to consume daily capsules. The doses of DHA prescribed daily were 120 [93], 300 [92], 400 [89,90], 600 [82,83], 800 [86,87,88], 1000 [91], 1020 [94], 1200 [80] and 2200 [84,85] mg per day. In one trial, women were provided with cereal bars to consume most days, with each cereal bar containing 240 mg/DHA [81]. Supplementation typically started mid-pregnancy and ended at birth [80,81,82,83,84,85,86,87,88,89,90]. Where supplementation continued after birth through infant formula and supplements for breastfeeding mothers, the intervention stopped at 30 days [93], 3 months [92], and 4 months of age [94]. Two trials excluded preterm-born infants from their follow-ups [84,85,92].

#### 3.2.2. Interventions for Neonates Born Preterm

Six RCTs with 1667 preterm infants administered a DHA intervention through sachets, oil or powders that could be added to milk, capsules for breastfeeding mothers, or fortified preterm infant formula [95,96,97,98,99,100,101,103,104,105]. Two trials compared a low or standard dose of DHA to a higher dose of DHA [98,99,100,103,104]. The majority of trials commenced their intervention within a week of birth, with the exception of one trial that commenced when the infants were 10–16 months of age [101]. Three RCTs ceased the intervention at discharge from hospital [95,98,99,100,103,104]. In one trial, infants were aged 9 months of age when the intervention ceased [96,97]; and in another trial, infants were 24 months of age [105]. The trial that delayed the intervention only commenced once formula feeding and breastfeeding had stopped [101]. The amount of DHA any individual infant received varied both within and between groups as dose was dependent on the amount of milk the infant was able to tolerate throughout the duration of the intervention.

#### 3.2.3. Postnatal Interventions for Breastfeeding Mothers

There were 2 eligible RCTs among 535 breastfeeding women [106,107]. A trial in Ethiopia (where dietary DHA intake is reported to be habitually low) randomized mother–infant pairs when infants were aged 6 to 12 months [106]. Mothers received capsules and infants received a corn–soy blend micronutrient complementary food supplement for 12-months [106]. Maternal capsules contained 215 mg DHA and the infant supplement provided 285 mg DHA [106]. Mother–infant pairs were randomized to 1 of 4 groups; (1) mothers’ capsules contained DHA but infant supplements did not, (2) mothers’ capsules and infant supplements both contained DHA, (3) infant supplements contained DHA but mothers’ capsules did not, and (4) neither capsules not the infant supplement contained DHA [106]. The other trial randomized infants within a week of birth and enrolled a reference group of mothers with habitually high dietary fish consumption [107]. Mothers were offered capsules as well as fortified muesli bars and cookies to provide 1.5 g LCPUFA [107].

#### 3.2.4. Postnatal Interventions Directly to Infants or Toddlers

Seven RCTs randomized 2205 infants and toddlers [108,109,110,111,112,113,114,115], the majority of which were exclusively term-born infants [108,109,110,111,112,113]. One trial included both formula-fed and breast-fed infants [113]. Five RCTs compared fortified infant formulas [108,109,110,111,112], one trial provided micronutrient complimentary food [115], and oil capsules that could be added to milk were provided in another [113]. Administered doses of DHA ranged from 0.12 to 0.96% of total fatty acids present in formula [108,109,110,111,112], or 250 to 280 mg DHA per day [113]. Supplementation went from 6 to 18 months of age in one trial [115], but most started within a week of birth in most trials and lasted 4–12 months [108,109,110,111,112,113].

### 3.3. Assessments of Behavioral Functioning and Behavior Problems

Behavioral functioning was assessed at a total of 43 different times among the 25 RCTs between 4 months and 20 years, with a total of 71 assessments and 401 group comparisons.

(a) Diagnosed behavioral problems (such as by a psychologist, psychiatrist or pediatrician) or use of prescription medication for a behavioral problem

Three trials explored differences in diagnosed behavioral problems or use of prescription medication for behavioral problems [87,88,91,100]. One study accessed national medical records to ascertain prescriptions of medications for treating behavioral problems and another accessed ADHD cases from a registry [91]. Parents reported diagnoses of behavioral problems in two trials [87,88,100]; and in one of these trials, parents additionally reported whether the child was taking any prescription medications for problem behavior in one study [99,100]. In this same study, parents were asked whether they had consulted a health care professional due to concerns for their child’s behavior [99].

(b) Clinician-administered general behavior measures and/or behavioral sub-scores of other measures

There were only two studies that included a clinician-administered measure of behavior [80,97]. A modified Wolke five-scale measure was used to assess behavior at 10 months in Bangladesh [80]. The Wolke scale has no overall score but five scales for activity, emotional tone, responsiveness to examiner, cooperation, and vocalization. In a preterm infant trial, the Behavioral Assessment of the Dysexecutive Syndrome for Children (BADSC) was administered when children reached 10 years of age [97].

A further nine studies involved a clinician-administered measure of another developmental domain that included a behavioral subscale or score [83,84,95,96,108,109,110,112,115]. The Bayley Scales of Infant Development (Bayley) is considered by many internationally to be the gold standard for assessing the development of infants and young children (up to ~3.5 years of age). There are multiple editions. The first and second editions includes a Behavioral Rating Scale (BRS) that assesses the child’s behavior in relation to complying with the instructions of the Bayley mental and motor assessments and was reported in four trials [83,108,109,110]. In one of these trials, the BRS scores were age standardized and classified as questionable if scores fell below the 25th centile, and classified as non-optimal if below the 10th centile [108]. The Griffiths Mental Development Scale (GMDS) is another global developmental test from birth to 8 years that includes a Personal–Social scale and was used in one trial at 2.5 years of age [84] and at 18 months of age in another trial [115]. The Knobloch, Passamanick and Sherrard’s Developmental Screening Inventory (KPSDSI) is a general developmental screening test that includes subscales measuring Adaptive, and Personal–Social behaviors. The KPSDSI was used in 2 preterm infant trials at 9 months of age [95,96] and in an infant trial at 9 months of age [112].

(c) Teacher-rated behavior measures

There was only one included trial that involved a teacher-rated measure of behavior. The Achenbach Child Behavior Checklist (CBCL) has eight empirically based syndrome scales (Aggressive Behavior, Anxious/Depressed, Attention Problems, Rule-Breaking Behavior, Somatic Complaints, Social Problems, Thought Problems, and Withdrawn/Depressed) and six DSM-oriented scales (Affective Problems, Anxiety Problems, Somantic Problems, ADHD Problems, Oppositional Defiant Problems and Conduct Problems). The teacher-rated, Dutch version of the CBCL was completed by teachers in one RCT in infants [111]. In an Australian trial, the Teacher Report Form (TRF) and the Gifted Rating Scale (GRS) were administered to school teachers when children were 6 years of age [114].

(d) Parent-rated behavior measures

There were eight studies that included a parent-rated measure of general behavior [84,85,86,87,88,90,92,100,102,111,113,114], an additional trial that included a parent-rated measure of development with a behavior score [93], and two trials that included parent-rated measures of temperament [81,99].

The Bayley-III includes optional age-standardized parent-rated Social–Emotional [S-E] and Adaptive-Behavior [A-B] scales, that were reported in two studies [86,92]. The Bayley-III S-E measures self-regulation, impactful use of emotions and engaging with others, and the A-B scale measures skills including self-care and self-direction, communication and social domains. The Strengths and Difficulties Questionnaire (SDQ) is a brief screen of general behavioral symptoms. The SDQ is summarized in an overall Total Difficulties score and includes subscales for emotional symptoms, hyperactivity, conduct problems, peer problems, and prosocial behavior. There is an additional Impact scale as an optional indication of whether or not the parent-perceived symptoms have a negative impact on the child’s quality of life. Overall scores range from 0 to 40 points, with higher scores indicating more problematic behavior (for all scores except the prosocial subscale). The SDQ was reported at 7 years of age in 3 studies [88,100,107] and at 3–5 years in two of these RCTs [87,99]. The Behavior Rating Inventory of Executive Functioning (BRIEF) measures behavioral manifestations of executive functions in the home environment. The BRIEF questionnaire has a version for children aged 5 to 18 years that has an overall Global Executive Composite score, two index scores for Behavioral Regulation Index, and Metacognition Index as well as subscale scores; Inhibit Scale, Shift Scale, Emotional Control Scale, Initiate Scale, Working Memory Scale, Plan/Organize Scale, Organization of Materials Scale, and Monitor Scale. The BRIEF was reported in two trials [88,100]. The BRIEF also has a version for children aged 2 to 6 years, the BRIEF-Preschool (BRIEF-P) that was used in 1 trial [87]. The BRIEF-P likewise includes an overall Global Executive Composite score, three index scores (Inhibitory Self-Control Index, Flexibility Index, and Emergent Meta-Cognition Index) and subscales Inhibition Scale, Shift Scale, Emotional Control Scale, Working Memory Scale, and Plan/Organize Scale. The BRIEF and BREIF-P scores are all scaled to a mean of 50 and SD 10, where a score greater than 64 is clinically indicative of dysfunction. The Conners 3rd Edition ADHD/DSM-IV Index-Parent (Conners 3^TM^ AI-P) measures Diagnostic and Statistical Manual of Mental Disorders vIV—defined symptoms of ADHD. Scores are age standardized to a mean of 50, SD 10, where a normal score is less than 60, and a score above 60 indicates more concerns than typical for a child of that age. The Conners was used in two trials [88,100]. The Brief Infant Toddler Social and Emotional Assessment (BITSEA) assesses socioemotional and behavioral problems in toddlerhood and was completed by parents in one of the preterm trials [102]. The BITSEA is comprised of seven scales (Competence, Problem, Externalizing, Internalizing, Dysregulation, Red Flag, ASD) which authors compared between groups as mean scores as well as dichotomized according to cut-off scores indicating a possible behavioral problem [102]. This same trial also asked parents to complete the Pervasive Developmental Disorders Screening Test-II (PDDST) to screen for ASD in children with developmental concerns and again compared both the mean score and proportion scoring over the cut off [102]. The Vineland Adaptive Behavior Scales (VABS) is a parent-completed questionnaire that measures adaptive behavior and was administered in one trial of preterm infants [105]. The CBCL includes a parent-rated version in addition to the teacher-version and was used in three trials [84,85,111,113,114]. One trial that used the parent-rated CBCL-derived seven scales (Emotionally Reactive, Anxious/Depressed, Somatic Complaints, Withdrawn, Sleep Problems, Attention Problems, and Aggressive Behavior) as well as the six DSM-oriented scales [113], whereas others reported just the overall score and subscales for internalizing, externalizing, and (for older children) competence [84,85]. The Behavioral Assessment System for Children (BASC)-2 has a Parent Rating Scale to capture adaptive and maladaptive behaviors in the home and community environment that was administered in two trials [83,90]. There is no overall score for the measure but scales for Externalizing Problems, Internalizing Problems, Adaptive Skills and Behavioral Symptoms [83,90]. In one of the trials, the Spanish version of the BASC-2 was adapted and administered through trained psychologists interviewing parents [90]. Infant Behavior Questionnaire (IBQ) is a parent-completed questionnaire for infants with a complimentary version for older children, the Early Childhood Behavior Questionnaire (ECBQ). There is no overall score, but each has several behavioral scales; the IBQ includes activity level, distress to normal stimuli, distress to limitations, soothability, smiling and laugher, duration of orienting. The ECBQ scales measure effortful control, and activity level. Both the IBQ and ECBQ were used in 1 preterm infant trial when children were 16–22 months of age [101] and the IBQ was used at 6 and 12 months of age in one trial in infants [108]. The Autism Spectrum Quotient: Children’s Version (AQ-Child) was administered to parents of 6-year-old children in one trial [114].

The Denver Developmental Screening Test (Denver) is a global infant and early childhood assessment that involves a combination of clinician-administered as well as parent-reported components. One postnatal trial adapted the Denver-II for use in Ethiopia and compared group scores on the Personal–Social subscale at enrollment into the trial, after 6 months on the intervention and again 12 months after enrollment [106]. The Ages and Stages Questionnaire (ASQ) is a parent-reported measure of global child development. The ASQ includes subscales to capture aspects of behavior, dependent on the child’s age. The ASQ was used in three trials, where the subscales reported were the Social–Emotional [S-E] subscale [106] or Personal–Social [S-P] subscale [93,103,104]. The Child Development Inventory (CDI) was another global measure that include a subscale for Social behavior. The CDI was adapted in Germany and used in one prenatal trial at 4 and 5 years of age [94].

Temperament was assessed via parent-ratings in two trials [81,99]. In one trial of preterm infants, parents completed the Short Temperament Scale for Children (STSC) when children were 3–5 years of age [99]. The STSC provides an overall score as well as subscales for approach, inflexibility, persistence, and rhythmicity. In one trial the Infant Temperament Questionnaire (ICQ) was completed by parents at 6 months to gain an overall score as well as subscales for fussy-difficult, unadaptable, dull, and unpredictable behaviors [81]. In this same trial, infants were followed up again at 12 months with the Revised Infant Temperament Questionnaire (RITQ) with another overall temperament score and, subscales for activity, rhythmicity, approachability, adaptability, intensity, mood, persistence, distractibility, and threshold [81].

(e) Self-reported behavior measures

There was only one self-reported assessment of behavior that was completed by the child themselves. The CBCL, which also includes a teacher-rated and a parent-rated version, includes a self-reported version that was used in one study when children were 12 years of age [85].

(f) Sensitivity and/or subgroup analyses

Subgroups or sensitivity analyses, where reported, were explored for birthweight [100,101], household income or SES [90,101], maternal intelligence [90], home environment [90], and sex [86,87,90,92,97,100,101,107,114].

### 3.4. Effect of DHA Intervention of Behavioral Functioning

#### 3.4.1. Maternal Interventions during Pregnancy

Of the 5913 pregnant women enrolled and randomized between 10 RCTs, there were behavioral assessments of 3476 (58.8%) children [80,81,82,83,84,85,86,87,88,89,90,91,92,93,94]. Assessments were conducted at 21 different ages between 4 months and 20 years of age. There were 28 measures of behavior, with a total of 220 comparisons between groups, including tests within subgroups and for interaction effects.

(a) Diagnosed behavioral problems (such as by a psychologist, psychiatrist or pediatrician) or use of prescription medication for a behavioral problem

Two prenatal trials explored the effect of a DHA intervention on clinically diagnosed behavioral problems [87,88,91]. A trial that accessed a registry for ADHD found no differences between their groups when the children were 14–20 years of age [91]. In one trial that collected parent-reported medically diagnosed behavioral problems, there were no differences in the proportion of ASD or ADHD at 4 years [87] or of any neurodevelopmental disorders at 7 years [88].

(b) Clinician-administered general behavior measures and/or behavioral sub-scores of other measures

There was only one prenatal study that included a clinician-administered measure of behavior [80] and two studies with clinician-administered developmental measures that included a behavioral subscale [83,84]. There were no group differences detected on the clinician-administered adaptation of the Wolke scale in 10-month-old children [80]. The BRS of the Bayley-II was reported by one prenatal study at 18 months and no effect of the DHA intervention was detected [83]. One trial included an objective measure of behavioral development as subscale of a global developmental assessment at 2.5 years, and detected no differences between the groups [84].

(c) Teacher-rated behavior measures

There were no reports of a teacher-rated measure of behavior.

(d) Parent-rated behavior measures

There were eight trials that involved a parent-rated measure of behavior [81,83,84,86,87,88,90,92,93,94] and many of these studies administered parent-rated measures at more than one time point [81,83,84,86,87,88,92,93].

One trial that reported using the Bayley-III to measure behavior at 4 months and at 12 months of age detected no group differences at either time point on either the S-E or A-B scale [92]. Another trial involved multiple measures of parent-rated behavior from infancy to school age [86,87,88]. At 18 months there was no effect of the DHA intervention on the Bayley-III S-E or A-B [86]. Nor was there a difference in the overall BRIEF-P score at 4 years, although overall scores of the SDQ were slightly poorer in the DHA group [86]. All subscales for the BRIEF-P (three indices and five scales) and SDQ (six subscales) were slightly poorer in the DHA group, although only the SDQ Hyperactivity scale, and the BRIEF-P Emergent Meta-Cognition Index and Plan/Organize Scale reached a statistically significant difference [86]. When the SDQ and BRIEF were completed at 7 years in this same sample, overall scores were worse in the DHA group, as was the Conners 3^TM^ AI-P and the 2 Index scores reported for the BRIEF [88]. Subscale scores were not reported for the SDQ or BRIEF in the 7 year follow-up [88]. Where scores differed between the groups in each of the comparisons, the magnitude was small (0.29 to 3.61 points) [87,88]. One prenatal trial administered the parent-rated CBCL with the overall score as well as subscales for internalizing and externalizing behavior at 2.5 years and again at 12 years of age [84,85]. At 2.5 years, authors additionally compared cut offs for scores indicative of a clinical problem [84] and at 12 years there was an additional Competence sub-score [85]. The authors detected no group differences in any of these comparisons [84,85]. The BASC-2 was administered at 26, 48, 60 and 72 months in one trial, with no group differences detected at any age [83]. The BASC-2 was administered at 5 years in one of the largest prenatal trials [90]. No group differences were reported for the scales for Externalizing Problems, Internalizing Problems, Adaptive Skills or Behavioral Symptoms [90].

The global ASQ screen was completed by parents at 4 and 6 months in one trial, and no effect of the DHA intervention was detected on the S-P subscale [93]. The CDI likewise revealed no group differences when the social subscale was compared at 4 and 5 years of age [94].

In the one trial that measured temperament with two measures, the ICQ at 6 months and the RITQ at 12 months, no differences were reported on overall or any subscale scores [81].

(e) Self-reported behavior measures

In the only trial that included a child self-report measure, administered at 12 years, there were no effects of the intervention detected on the overall CBCL score, nor the internalizing, externalizing or competency sub-scores, although the sample was small with only 50 children included [85].

(f) Sensitivity and/or subgroup analyses

Of the 10 prenatal RCTs, 3 reported conducting any subgroup analyses [86,87,88,90,92], for sex [86,87,88,90,92], maternal intelligence [90] and stimulation in the home environment as well as socioeconomic status [90]. There were no interactions with sex detected at a 5-year follow-up [90]. Subgroup analyses for sex on the parent-rated Bayley-III Social–Emotional and adaptive behavior scores at 4 and 12 months did not reveal any sex by treatment interactions [92]. One large trial showed that although there were no overall differences in parent-rated behavior at 18 months [86], there was evidence that DHA group girls had a lower (poorer) adaptive behavior scores [86]. The 4-year follow-up of these children revealed no sex by treatment interaction [87] and the subgroup analyses were not reported for the 7-year behavioral assessments [88]. Only one trial explored an interaction effect for quality of stimulation in the home environment during childhood or socioeconomic status, with no null effects found [90]. Nor was there any interaction between DHA intervention and maternal intelligence [90].

#### 3.4.2. Interventions for Neonates Born Preterm

Of the 1667 preterm infants enrolled and randomized, there were behavioral assessments of 1402 (84.1%) children [95,96,97,98,99,100,101,102,103,104,105]. Assessments were conducted at 10 different ages from 6 months and 10 years of age. There were 19 measures of behavior, with a total of 116 comparisons between groups, including tests within subgroups and for interaction effects.

(a) Diagnosed behavioral problems (such as by a psychologist, psychiatrist or pediatrician) or use of prescription medication for a behavioral problem

Although infants born preterm have an increased risk of developing behavioral problems compared with infants born at term, diagnosed problems or use of medications was only collected in one large trial [99,100]. More parents from the high-DHA group had consulted a health care professional due to concerns for their child’s behavior, although no children were taking any prescription medications for problem behavior at the time of the follow-up (3–5 years of age) [99]. At 7 years of age in this cohort, there were no differences in diagnoses of ADHD, ASD, and no differences in the use of ADHD medications [100].

(b) Clinician-administered general behavior measures and/or behavioral sub-scores of other measures

There were two trials with an assessment of behavior performed by a clinician [95,96,97]. One trial administered the BADSC at 10 years and found no difference in performance between the groups [97]. Two trials both used the KPSDSI screening test at 9 months of age which includes two subscales for Adaptive behavior and Personal–Social behaviors [95,96]. Neither test detected a difference [95,96].

(c) Teacher-rated behavior measures

None of the trials included a teacher-rated measure of behavioral functioning.

(d) Parent-rated behavior measures

Parent-rated measures of behavior were included in three of the six trials in preterm samples [99,100,101,102,105], and one included global measures with a parent-rated behavioral subscale [103,104]. One follow-up of a small subset from the larger trial included a parent-rated assessment of temperament [99]. One trial used the VABS-II to assess adaptive behavior at 12 and 24 months, and found no effect of the DHA intervention [105]. In one large trial, no group differences were detected on mean overall or subscale scores of the SDQ in a subset of the sample at 3–5 years of age [99]. When scores on the SDQ were categorized as normal or abnormal, there were no group differences [99]. When the SDQ was administered again in the larger sample at 7 years, there were no group differences on overall or subscale scores [100]. Nor was there an effect on the BRIEF or Conners 3^TM^ AI-P [100]. In a trial that supplemented preterm infants after weaning, parent-rated behavior on the IBQ-R/ECBQ at 16–22 months detected no group differences in effortful control or activity level [101]. Parents in this same trial also completed the BITSEA and the PDDST-II at the same time, and no differences in any of the mean group scores were detected [102]. When dichotomized by cut-off scores for clinical concern, there was no effect on found on the seven BITSEA scales, but treatment group children were slightly less likely to have scored in the clinically concerning range on the PDDST-II than the control group children [102].

The ASQ (a measure of general development) was completed by parents at 6 months and again at 20 months and the ASQ S-P subscale was compared between groups, with no differences detected [103,104].

In the 1 trial that included a measure of temperament, there was 1 report of a negative effect on 1 of 4 subscales of the STSC, and no effect on the overall score of the STSC in one trial at 3–5 years of age [99]. The Persistence subscale at 3–5 years of age was slightly poorer in the DHA group [99]. When STSC scores were categorized as normal or abnormal there were no group differences [99].

(e) Self-reported behavior measures

There were no trials that included a self-reported measure of behavioral functioning.

(f) Sensitivity and/or subgroup analyses

Of the six preterm infant RCTs, half reported conducting a subgroup analysis [97,100,101,102]. Subgroup analyses were conducted for sex [97,100,102], birthweight [100,101], and household income [101]. One of the earlier RCTs found no sex by treatment interaction on behavior at 10 years [97]. A recent trial identified no sex by treatment interaction at 16–22 months of age for the majority of outcomes measured, and one instance where a benefit of the intervention was seen in symptoms of ASD among girls only, and one instance where there was a benefit of the intervention on symptoms of ASD in boys only [101,102]. In a larger trial including both breastfed and formula-fed preterm infants, subgroup analyses revealed a sex by treatment interaction on behavior at 7 years of age [100]. On the Conners 3^TM^ AI-P and the BRIEF, girls in the DHA group had consistently slightly poorer scores for all outcomes, and the difference reached statistical significance on the overall score as well as the Behavioral Regulation Index, Inhibit Scale, and Monitor Scale [100]. Of the seven outcomes for the SDQ, girls similarly had consistently worse scores but only the conduct problems subscale reached statistical significance [100]. Birthweight <1250 g or >1250 g did not appear to interact with DHA in this same trial for any of these behavioral outcomes [100]. Nor was there an interaction between birthweight and DHA treatment in a smaller trial [101]. This trial also explored household income and found an interaction effect with DHA supplementation where effortful control was slightly poorer in the DHA group as household income increased [101].

#### 3.4.3. Postnatal Interventions for Breastfeeding Mothers

Of the 535 breastfeeding women enrolled and randomized, there were behavioral assessments of 424 (79.3%) children [106,107]. Assessments were conducted at 4 different ages between 6 months and 7 years of age. There were 7 measures of behavior, with a total of 18 comparisons between groups.

(a) Diagnosed behavioral problems (such as by a psychologist, psychiatrist or pediatrician) or use of prescription medication for a behavioral problem

There were no explorations of diagnosed behavioral problems in either maternal postnatal intervention trial.

(b) Clinician-administered general behavior measures and/or behavioral sub-scores of other measures

Neither postnatal trial employed a clinician-administered measure of behavior.

(c) Teacher-rated behavior measures

None of the maternal postnatal intervention trials used a teacher-rated measure of behavior.

(d) Parent-rated behavior measures

Both postnatal trials involved parent-rated measures [107]. In one trial, the SDQ was used at 7 years and revealed no differences on Total Difficulties Score or the 5 subscale scores [107]. The trial in Ethiopia detected no differences on the Denver-II subscale for Personal–Social or ASQ S-E subscale [106].

(e) Self-reported behavior measures

Neither trial included a self-reported measure of behavior.

(f) Sensitivity and/or subgroup analyses

One postnatal trial tested for a sex by treatment interaction effect at 7 years of age [107]. Whilst the majority of SDQ scores revealed no group differences or interactions, the Prosocial Behavior Scale revealed an interaction in which treatment group boys had poorer scores than control group boys [107]. Scores did not differ between treatment group and control group girls [107].

#### 3.4.4. Postnatal Interventions Directly to Infants or Toddlers

Of the 2205 infants enrolled and randomized into a RCT of infants or toddlers, there were behavioral assessments of 1565 (71.0%) children [108,109,110,111,112,113,114,115]. Only one trial included infants that were breastfed [113] and the majority included only term-born infants [108,109,110,111,112,113]. Assessments were conducted at 9 different ages between 6 months and 6 years of age. There were 17 measures of behavior, with a total of 47 comparisons between groups, including tests within subgroups and for interaction effects.

(a) Diagnosed behavioral problems (such as by a psychologist, psychiatrist or pediatrician) or use of prescription medication for a behavioral problem

None of the RCTs supplementing infants or toddlers with DHA explored whether there was an effect on diagnosed behavioral problems or receipt of medications used to treat behavioral problem.

(b) Clinician-administered general behavior measures and/or behavioral sub-scores of other measures

There were three trials in infants that included a clinician-administered measure of behavior [108,109,110] and one clinician-administered developmental measure that included a behavioral subscale. The BRS score of the Bayley-II was compared between groups at 6 months of age and again at 12 months of age in one trial [108] and at 18 months in two other trials [109,110]. No group differences were detected at any age in any trial [108,109,110]. The KPSDSI at 9 months revealed no effect of DHA intervention [112] and nor did the GMDS at 18 months of age [115].

(c) Teacher-rated behavior measures

There were two trials that included a teacher-rated measure of behavior [111,114]. One trial provided teachers with the CBCL when the children were 9 years old [111]. No differences were detected between the groups [111]. An alternate trial asked teachers to complete the TRF and GRS at 6 years, and likewise found no effect of DHA supplementation [114].

(d) Parent-rated behavior measures

There were three trials in infants that included one or more parent-rated measures of behavior [108,111,113,114]. The IBQ at 6 and at 12 months of age revealed no effect of DHA intervention on 5 of 6 subscale scores at both ages [108]. The smiling and laughter score was slightly but statistically significantly poorer for the DHA group when compared with the control group [108]. The Bayley-III S-E and A-B subscales were administered in 1 trial when children were 18 months of age and no group differences were detected [113]. The CBCL provided to parents when their children were 9 years of age in one trial [111], and at 18 months [113], and 6 years of age in another trial [114]. Comparisons revealed no effect of DHA intervention on the overall CBCL scores [111,113,114]. When 13 CBCL subscales were compared at 18 months, 12 were null whilst the anxious/depressed subscale was slightly worse in the DHA groups, even though the Bayley-III measures at the same time point were null [113]. When these same children were followed up at 6 years and 3 subscales were compared between groups, 2 showed no effect of DHA and the externalizing behavior scale was worse in the DHA group which appeared to be driven by the oppositional defiance sub-scale [114]. No differences were found in internalizing behavior or Total behavior at 6 years [114]. Nor were differences detected on the AQ-Child in this same trial at 6 years of age [114].

(e) Self-reported behavior measures

None of the trials in infants asked children to rate their own behavioral functioning.

(f) Sensitivity and/or subgroup analyses

Of the seven trials conducted during infancy, there was only one that reported conducting a subgroup analysis, for a sex by treatment interaction [114]. A sex by treatment interaction was detected in which DHA group boys had higher externalizing behavior and oppositional defiance than boys in the control group [114].

## 4. Discussion

This review is the first to focus on the effect of DHA supplementation in the first 1000 days on child behavioral functioning. At the time of this review, no study included behavioral functioning as a primary outcome or a main outcome of interest. Recent Cochrane reviews of DHA interventions in the first 1000 days identified a total of 126 DHA RCTs (70 in pregnancy [69], 17 in preterm infants [70], 8 to breastfeeding women [71], and 31 in infants and toddlers [72]). However, only 25 of these were eligible for inclusion in the current review due to the absence of child behavior as an outcome in the vast majority (80%) of intervention trials. Behavior, when measured, was generally a secondary or exploratory outcome, and findings are subject to type 1 errors.

There were no effects of a DHA intervention detected on behavior problems, or overall behavioral scores or subscale scores for 19 included RCTs [80,81,82,83,84,85,89,90,91,92,93,94,95,96,97,103,104,105,106,109,110,111,112,115]. Contrary to expectations, only one study identified a potential beneficial effect of DHA supplementation, on 3 out of 55 behavioral group comparisons [101,102]. There were six studies (one prenatal RCT [86,87,88], two preterm neonate trials (including the one that found a potential benefit) [98,99,100,101], one in breastfeeding women [107], and two directly in infants [108,113,114]) in which one or more comparisons (total 23) indicated an adverse effect of DHA intervention on a behavioral outcome. Importantly, in all of these studies, there were multiple behavioral outcomes and the majority of behavioral comparisons were null [86,87,88,98,99,100,101,107,108,113,114]. Of the larger 2 trials, there was 6 instances of a worse score in the DHA group out of 43 comparisons in one [86,87,88], and 8 instances of a worse score from 59 comparisons in the other [99,100]. Most differences in the latter trial were within girls only [99,100], whilst two other studies found that it was boys who had poorer scores after supplementation with DHA [107,113,114]. Few studies reported diagnosed behavioral problems, and those that did found no group differences. All reported differences were in parent-rated measures, although admittedly the majority of measures were parent-rated. However, given the prevalence of diagnoses for behavioral problems and the sample sizes of included studies, a difference in the proportion of diagnosed problems is unlikely to be detected as most studies were underpowered.

Between the 25 RCTs included in our review, there were 71 assessments of behavior administered, and 401 group comparisons of a behavioral outcome, meaning that for each behavioral assessment, there was an average of 5 comparisons. The heterogeneity of the behavioral assessments, study characteristics and group comparisons conducted prevented combining the data in a meta-analysis. Measures of behavior typically involve an overall score made up of several subscale scores that reflect specific, individual aspects of behavior, such as hyperactivity or impulsiveness. Exploration of individual domains of behavior is important for discerning whether DHA effects behavior globally or particular aspects of behavior only, although this does further increase the risk of a type 1 error. Furthermore, of the 10,032 participants enrolled in these trials, behavioral outcomes were reported for only 6867 children, equating to an overall average drop-out rate of almost 35%, meaning that attrition bias is likely [118]. The largest losses to follow-up were from the trials with a maternal intervention during pregnancy (58%) possibly due to post-randomization exclusion of infants born preterm by some trials, whilst trials conducted in preterm infants had the highest retention (84%). Furthermore, there was evidence of publication bias, where results of one follow-up were available only as an abstract from conference proceedings [91], and results of another study were available as a doctoral dissertation [81].

Of the two potentially eligible trials that were excluded, one found no effect of the egg intervention on Personal–Social behavior [116] and the other reported improvements in some but not all behavioral scores after supplementation for 31 preterm-born children who displayed symptoms of ASD [117]. Including these studies in the review would not have impacted our conclusion.

DHA is widely thought to be beneficial for brain development. There is currently no clear mechanism for DHA supplementation to cause harm, although it may be that excess DHA is detrimental. A recent RCT found that providing high-dose DHA to pregnant women with low omega-3 long-chain polyunsaturated fatty acid status was beneficial for protecting against early preterm birth (<34 weeks’ gestation), but in women with higher omega-3 long-chain polyunsaturated fatty acid status, high-dose DHA intervention appeared to increase the risk of early preterm birth [119]. Among the very and extremely preterm infant population (<29 weeks’ gestation at birth), new evidence is emerging that high-dose DHA supplementation may increase the risk of the serious lung condition, bronchopulmonary dysplasia [120,121]. In animal models, suggested adverse effects of DHA have included a shortened lifespan in mice on a long-term fish oil diet, apparently due to oxidative stress and decreased cellular function [122] as well as detrimental proliferation of neural stem progenitor cells in rats fed high-dose DHA [123]. However, the potential of these suggested mechanisms to account for adverse effects of DHA supplementation detected in human studies needs to be established. It is also possible that behavioral development is particularly sensitive to exposure to nutrients in excess of needs during fetal development, as has been seen with other nutrients such as iron exposure in pregnancy [124].

Our review of the effect of a DHA intervention in the first 1000 days on behavioral development has a similar conclusion to reviews of DHA supplementation over the same period on cognition, motor or visual development [76], or language abilities [78]. Additionally, several reviews and meta-analyses of more specific windows within the first 1000 days have identified little, if any evidence of benefits of DHA supplementation for brain development [68,69,70,71,72,74,125]. Our previous review of DHA interventions and subsequent language abilities revealed that whilst the majority of language measures were null, four suggested a benefit and two suggested an adverse effect [78]. Differences in findings between RCTs of DHA supplementation and observational studies of dietary sources of DHA could be due to a number of reasons, such as additional nutrients present in foods naturally rich in DHA and the inherent confounding in observational studies due to the difficulty in adjusting for complex confounding factors [67]. For example, several factors found to be associated with low fish consumption such as being a single parent, lower levels of maternal education, high level of family adversity, crowding in the family home and not being a homeowner as well as poor lifestyle factors such as not breastfeeding, low parenting scores and smoking [16] are also considered to influence child developmental outcomes. Furthermore, there is evidence to suggest that cohort studies have a tendency to inflate positive effects compared with RCTs. One notable study found 56% of non-randomised trials found a positive treatment effect compared with 30% of RCT [126] and this finding has been replicated in other studies [126,127,128,129].

We have determined that the existing evidence does not conclusively support or refute the hypothesis that DHA supplementation in the first 1000 days of life improves children’s behavioral functioning, but that it may in fact adversely affect behavior. Our interpretation should be considered in light of the limitations of the body of literature, and any potentially negative effect would need to be verified in future research. Whilst it would be unethical to commence a new intervention with DHA in the first 1000 days specifically to determine adversity, we strongly recommend that behavioral assessments be added as outcomes to new trials, and to follow-up studies for existing trials. Many behavioral assessments are quick and easy to administer with versions for parent and teachers to complete, as well as self-report for literate children. The SDQ for example is a readily available behavioral screening tool that takes approximately 5 min to complete.

## 5. Conclusions

Although there is a plethora of DHA interventions in the first 1000 days, only a small proportion (less than one-quarter) appear to include a measure of child behavior. This review is the first attempt to synthesize the results of these behavioral outcomes. Of the 71 behavioral measures, there were 401 comparisons reported in the included studies, and the vast majority detected a null effect. There were 6 trials with 23 parent-rated outcomes that highlighted a potentially adverse effect of DHA on behavior, and one of these trials also detected a potential benefit of DHA on behavior. No differences were reported in the prevalence of clinically diagnosed behavioral problems such as ASD or ADHD. However, at present, the evidence is insufficient to reach a definitive conclusion regarding the effect of early DHA interventions on child behavior. Given the paucity of evidence, we do not recommend that pregnant women, infants or young children consume DHA supplements in order to improve behavioral functioning. This review highlights the need for future RCTs, as well as future follow-ups of existing RCTs to prioritize child behavior as an outcome.

## Figures and Tables

**Table 1:** Summary of the characteristics of randomized controlled trials and the results of the behavioral assessments.

Author and Reference	Setting Country; Place of Recruitment	Participants Sample Randomized Sample Characteristics	Intervention Duration, Form, Treatment and Control Intervention	Assessment Age, n Outcome Measure	Result
Maternal Prenatal Interventions					
Tofail, 2006 [80]	Bangladesh; house-to-house survey	*N* enrolled: 400 Trt:200, ctrl:200 Third-world setting with endemic poverty, illiteracy, poor hygiene, overcrowding and poor housing	Duration: 25 week preg to birth Form: 4 capsules daily Trt: n-3 3000 mg/day, DHA 1200 mg/day Ctrl: soy oil	Age: 10 mo, *n* = 249	
				Wolke	
				-scores: 5	No diff
Judge, 2006 [81]	USA; hospital	*N* enrolled: 48 Trt:27, ctrl:21	Duration: 24 week preg to birth Form: cereal bar Trt: n-3240 mg/bar, average DHA 240 mg/day Ctrl: corn oil	Age: 6 mo, *n* = 38	
				ICQ	No diff
				-sub-scores: 4	No diff
				Age 12 mo, *n* = 28	
				RITQ	No diff
				-sub-scores: 9	No diff
Dunstan, 2008 [84]; Meldrum, 2015 [85]	Australia; antenatal clinic	*N* enrolled: 98 Trt:52, ctrl:46 All had allergic disease Excluded: normal diet includes >2 fish meals/week	Duration: 20 week preg to birth Form: 4 capsules daily Trt: n-3 3300 mg/day, DHA 2200 mg/day Ctrl: olive oil	Age: 2.5 years, *n* = 71	
				GMDS	No diff
				CBCL	No diff
				-sub-scores: 2	No diff
				Age: 12 years, *n* = 50	
				CBCL parent	No diff
				-sub-scores: 3	No diff
				CBCL child	No diff
				-sub-scores: 3	No diff
Makrides, 2010 [86]; Makrides, 2014 [87]; Gould, 2017 [88]	Australia; antenatal clinic	*N* enrolled: 2399 Trt:1197, ctrl:1202 Singletons Subset for neurodevelopmental follow-up *n* = 726 (preterm and randomly selected term)	Duration: 18–21 week preg to birth Form: 3 capsules daily Trt: 800 mg DHA/day Ctrl: vegetable oil	Age: 18 mo, *n* = 726	
				Bayley-III S-E	No diff
				-subgroup: girls/boys	No diff
				Bayley-III A-B	No diff
				-subgroup: girls/boys	Trt girls worse
				Age: 4 years, *n* = 646	
				SDQ	Trt worse
				-sub-scores: 6	1—Trt worse, 5—No diff
				-subgroup: girls/boys	No diff
				BRIEF-P	No diff
				-sub-scores: 8	2—Trt worse, 6—No diff
				-subgroup: girls/boys	No diff
				Diagnoses	No diff
				Age: 7 years, *n* = 543	
				SDQ	Trt worse
				BRIEF	Trt worse
				-sub-scores: 2	2—Trt worse
				Conners 3^TM^ AI-P	Trt worse
				Diagnoses	No diff
Ramakrishnan, 2010 [89]; Ramakrishnan, 2016 [90]	Mexico; antenatal clinic	*N* enrolled: 1094 Trt:547, ctrl:547 Medium–low SES Excluded: if taking *n*-3	Duration: 18–22 weeks preg to birth Form: 2 capsules daily Trt: 400 mg/day DHA Ctrl: olive oil	Age: 5 years, *n =* 797	
				BASC-2	No diff
				-scores: 4	No diff
				-sub-scores: 19	No diff
				-subgroup: girls/boys	No diff
				-subgroup: maternal intelligence	No diff
				-subgroup: SES	No diff
				-subgroup: home environment	No diff
Carlson, 2013 [82]; Colombo, 2019 [83]	USA; antenatal clinics	*N* enrolled: 350 Trt:178, ctrl:172 Singleton, healthy, normal BMI	Duration: mean 14.5 weeks preg to birth Form: 3 daily capsules Trt: 600 mg/day DHA Ctrl: soy and corn oil	Age: 18 mo, *n =* 186	
				Bayley-II BRS	No diff
				Age: 36 mo, *n =* 141	
				BASC-2	
				-scores: 4	No diff
				Age: 48 mo, *n =* 140	
				BASC-2	
				-scores: 4	No diff
				Age: 60 mo, *n =* 140	
				BASC-2	
				-sub-scores: 4	No diff
				Age: 72 mo, *n =* 140	
				BASC-2	
				-sub-scores: 4	No diff
Strom, 2013 [91]	Denmark; antenatal clinic	*N* enrolled: NR Trt:NR, ctrl:NR Singletons	Duration: NR Form: capsules Trt: 1 g DHA/day Ctrl: NR	Age: 14–20 years, *n =* 1051	
				ADHD cases from registry	No diff
Miller, 2016 [92]	USA; antenatal clinic	*N* enrolled: 115 Trt:60, ctrl:55 Singletons	Duration: 24–28 weeks preg to 3 mo Form: capsules Trt: 300 mg DHA + 67 mg EPA/day Ctrl: sunflower oil	Age: 4 mo, *n =* 91	
				Bayley-III S-E	No diff
				-subgroup: girls/boys	No diff
				Bayley-III A-B	No diff
				-subgroup: girls/boys	No diff
				Age: 12 mo, *n =* 83	
				Bayley-III S-E	No diff
				-subgroup: girls/boys	No diff
				Bayley-III A-B	No diff
				-subgroup: girls/boys	No diff
Ostradrahimi, 2017 [93]	Iran; health care centres	*N* enrolled: 150 Trt:75, ctrl: 75	Duration: 20 weeks preg to 30 days Form: capsules Trt: 120 mg DHA + 180 mg EPA/day Ctrl: liquid paraffin	Age: 4 mo, *n =* 148	
				ASQ S-P	No diff
				Age: 6 mo, *n =* 146	
				ASQ S-P	No diff
Brei, 2017 [94]	Germany; NR	*N* enrolled: 208 Trt:104, ctrl:104	Duration: 15 weeks preg to 4 mo Form: capsules (+ dietary counselling to lower AA intake) Trt: 1020 mg DHA + 180 mg EPA/day Ctrl: general dietary information	Age: 4 years, *n =* 119	
				CDI	No diff
				Age: 5 years, *n =* 130	
				CDI	No diff
Interventions for Preterm (Born <37 Weeks’ Gestation) Neonates					
Fewtrell, 2002 [95]	UK; neonatal units	*N* enrolled: 195 Trt:95, ctrl:100 <37 week, birthweight <1750 g, FF	Duration: <10 days until discharge Form: formula Trt: LCPUFA formula Ctrl: standard formula	Age: 9 mo, *n =* 158	
				KPSDSI	
				-score: 2	No diff
Fewtrell, 2004 [96]; Isaacs, 2011 [97]	UK; neonatal units	*N* enrolled: 238 Trt:122, ctrl:116 <35 week, birthweight ≤ 2000 g FF	Duration: before discharge to 9 mo CA Form: formula Trt: LCPUFA formula Ctrl: standard formula	Age: 9 mo C,A *n =* 117	
				KPSDSI	No diff
				Age: 10years, *n =* 107	
				BADSC	No diff
				-subgroup: girls/boys	No diff
Henriksen, 2008 [103]; Westerberg, 2011 [104]	Norway; neonatal units	*N* enrolled: 141 Trt:68, ctrl:73 Birthweight<1500 g BF only	Duration: from enteral feeds to discharge from hospital or infant finished the 100 mL bottle of oil Form: oil added to breastmilk Trt: 32 mg DHA + 31 mg AA/100 mL breastmilk Ctrl: soy oil	Age: 6 mo CA, *n =* 105	
				ASQ S-P	No diff
				Age: 20 mo CA, *n =* 92	
				ASQ S-P	No diff
Makrides, 2009 [98]; Smithers, 2010 [99]; Collins, 2015 [100]	Australia; neonatal units	*N* enrolled: 657 Trt:322, ctrl:335 Singletons and multiples <33 week Small subset for follow-up at 26 mo FF and BF	Duration: <5 days of starting full enteral feeds to term equivalent Form: preterm infant formula, 6 capsules daily to breastfeeding mothers Trt: formula 1% DHA Ctrl: formula 0.35% DHA	Age: 3–5 years CA, *n =* 125	
				SDQ	No diff
				-sub-scores: 5	No diff
				STSC	No diff
				-sub-scores: 5	1—Trt worse, 5—No diff
				Health care consultation	Trt worse
				Prescription medication	No diff
				Age: 7 years CA, *n =* 604	
				SDQ	No diff
				-sub-scores: 6	No diff
				-subgroup: girls/boys	1—Trt girls worse, 5—No diff
				-subgroup: birthweight <1250 g/≥1250 g	No diff
				BRIEF	No diff
				-sub-scores: 10	No diff
				-subgroup: girls/boys	5—Trt girls worse, 6—No diff
				-subgroup: birthweight <1250 g/≥1250 g	No diff
				Conners 3^TM^ AI-P	No diff
				-subgroup: girls/boys	Trt girls worse
				-subgroup: birthweight <1250 g/≥1250 g	No diff
				Diagnoses	No diff
				Prescription medication	No diff
Keim, 2018 [101,102]	USA; neonatal intensive care units	*N* enrolled: 377 Trt:189, ctrl:188 Singletons and multiples <35 week, no longer FF or BF	Duration: 10–16 mo CA for 6 mo Form: dissoluble powder Trt: 200 mg DHA + 200 mg AA/day Ctrl: 400 mg corn oil/day	Age: 16–22 mo, *n =* 377	
				IBQ-R/ECBQ	
				-scores: 2	No diff
				-subgroup: higher household income	1—Trt worse, 1—No diff
				-subgroup: girls/boys	No diff
				-subgroup: birthweight <1250 g/≥1250 g	No diff
				BITSEA	
				-scores: 14	No diff
				-subgroup: girls/boys	No diff
				PDDST-II	13—No diff, 1—Trt girls better
				-scores: 2	1—No diff, 1—Trt better
				-subgroup: girls/boys	1—No diff, 1—Trt boys better
Andrew, 2018 [105]	UK; neonatal units	*N* enrolled: 59 Trt:29, ctrl:30 Singletons <31 week/with risk of neurodevelopmental impairment (such as brain injury)	Duration: from full milk feeds for 2 years Form: sachet to mix with milk or food Trt: DHA 1% fatty acids Ctrl: no DHA	Age: 12 mo, *n =* 41	
				VABS-II	No diff
				Age: 24 mo, *n =* 41	
				VABS-II	No diff
Postnatal Interventions for Breastfeeding Mothers					
Cheatham, 2011 [107]	Denmark; antenatal GP visit (via Danish National Birth Cohort)	*N* enrolled: 175 Trt:62, ctrl:60, Ref:53 Healthy term infants BF Habitual fish intake below Danish median	Duration: <7days for 4 mo Form: muesli bars, cookies and capsules Trt: 4.5 g fish oil, 1.5 g LCPUFA Ctrl: olive oil Ref: high habitual fish intake	Age: 7 years, *n =* 98	
				SDQ	No diff
				-sub-score:5	No diff
				-subgroup: girls/boys	5—No diff, 1—Trt boys worse
Argaw, 2018 [106]	Ethiopia; NR	*N* enrolled: 360 Trt1:90, Trt2:89, Trt3:90, ctrl:91 BF healthy singletons	Duration: 6–12 mo for 12 mo Form: mother—capsules, child—complimentary food supplements Trt1:mother—215 mg DHA + 285 mg EPA, child—169 mg DHA + 331 mg EPA Trt2:mother—215 mg DHA + 285 mg EPA Trt3: child—169 mg DHA + 331 mg EPA ctrl: mother—corn oil, child—corn + soy oil	Age: baseline, 6–12 mo, *n =* NR	
				Denver	No diff
				ASQ S-E	No diff
				Age: after 6 mo, 12–18 mo, *n =* 326	
				Denver	No diff
				ASQ S-E	No diff
				Age: after 12 mo, 18–24 mo, *n =* 313	
				Denver	No diff
				ASQ S-E	No diff
Postnatal Interventions Directly to Infants and Toddlers					
Auestad, 2001 [108]	USA; children’s hospital	*N* enrolled: 404 Trt1:82, Trt2:80, ctrl:77, BF:165 Healthy term born FF	Duration: <7 days to 12 mo Form: formula Trt1: 0.13% DHA—egg Trt2: 0.13% DHA—fish/fungal Ctrl: no LCPUFA BF: Trt1 and Trt2 formula if stopped BF	Age 6 mo, *n =* 239	
				IBQ	
				-scores: 6	1—Trt worse, 5—No diff
				Bayley-II BRS	No diff
				Age 12 mo, *n =* 239	
				IBQ	
				-scores: 6	1—Trt worse, 5—No diff
				Bayley-II BRS	No diff
Lucas, 1999 [112]	UK; hospitals	*N* enrolled: 447 Trt:155, ctrl:154, BF:138 Term born FF	Duration: <7 days to 6 mo Form: formula Trt: 0.32% DHA Ctrl: no DHA	Age: 9 mo, *n =* 241 + BF = NR	
				KPSDSI	No diff
Birch, 2000 [109]	USA; hospitals	*N* enrolled: 119 Trt1:26, Trt2:27, ctrl:26, BF:40 Healthy term born FF	Duration: <5 days to 17 weeks Form: formula Trt1: 0.35% DHA Trt2: 0.36% DHA Ctrl: no DHA BF: no formula	Age: 18 mo, *n =* 76	
				Bayley-II BRS	No diff
de Jong, 2012 [111]	Netherlands; antenatal clinics	*N* enrolled:474 Trt:145, ctrl:169, BF:160 Healthy term born FF	Duration: 2 mo to 6 mo Form: formula Trt: 0.3% DHA + 0.45% AA Ctrl: no DHA BF: Trt formula if stopped BF	Age: 9 years, *n =* 341	
				CBCL-Parent	No diff
				CBCL-Teacher	No diff
Drover, 2011 [110]	USA; hospitals	*N* enrolled: 159 Trt1:38, Trt2:39, Trt3:40, ctrl:42 Healthy full-term singletons Low SES FF	Duration: 1–9 days to 12 mo Form: formula Trt1: 0.32% DHA Trt2: 0.64% DHA Trt3: 0.96% DHA Ctrl: no DHA	Age: 18 mo, *n =* 92	
				Bayley-II BRS	No diff
Phuka, 2012 [115]	Malawi; rural community with prevalent stunting	*N* enrolled: 182 Trt1:61, Trt2:61, Ctrl:60	Duration: 6 mo to 18 mo Form: micronutrient complementary food Trt1: 50 g micronutrient-fortified lipid spread; DHA dose NR Trt2: 25 g micronutrient-fortified lipid spread, DHA dose NR Ctrl: micronutrient-fortified corn–soy flour	Age 18 mo, *n =* 163	
				GMDS	No diff
Meldrum, 2012 [113]; Meldrum, 2020 [114]	Australia; antenatal clinic	*N* enrolled: 420 Trt:218, ctrl:202 All mothers had allergic disease FF and BF Excluded preterm	Duration: birth to 6 mo Form: oil capsules Trt: 250–280 mg/day DHA Ctrl: olive oil	Age:18 mo, *n =* 413	
				Bayley-III S-E	No diff
				-sub-score: 1	No diff
				Bayley-III A-B	No diff
				CBCL (*n =* 269)	No diff
				-sub-score: 13	12—No diff,1—Trt worse
				Age: 6 years, *n =* 303	
				AQ-Child	No diff
				CBCL	No diff
				-sub-scores: 3	2—No diff, 1—Trt worse
				-subgroup: girls/boys	Trt boys worse
				TRF	No diff
				GRS, *n =* 66	No diff

AA = Arachidonic Acid. AQ-Child = Autism Spectrum Quotient: Children’s Version. ASQ = Ages and Stages Questionnaire (subscale: Social–Emotional (S-E) or Personal–Social (S-P)). BASC = Behavioral Assessment System for Children (no overall score, scales: Externalizing Problems, Internalizing Problems, Adaptive Skills and Behavioral Symptoms). BADSC = Behavioral Assessment of the Dysexecutive Syndrome for Children. Bayley = Bayley Scales of Infant Development (edition III has optional parent-rated Social–Emotional (S-E) and Adaptive-Behavior (A-B) scales, editions I and II have BRS = Behavior Rating Scale). BF = breastfed. BITSEA = Brief Infant Toddler Social and Emotional Assessment (toddlers, no overall score, scales: Competence, Problem, Externalizing, Internalizing, Dysregulation, Red Flag, ASD). BRIEF-P = Behavior Rating Inventory of Executive Functioning (children aged 2 to 6 years, Global Executive Composite overall, subscales: Inhibitory Self-Control Index, Flexibility Index, Emergent Meta-Cognition Index, Inhibition Scale, Shift Scale, Emotional Control Scale, Working Memory Scale, Plan/Organize Scale). BRIEF = Behavior Rating Inventory of Executive Functioning (children aged 5 to 18 years, Global Executive Composite overall, subscales: Behavioral Regulation Index, Metacognition Index, Inhibit Scale, Shift Scale, Emotional Control Scale, Initiate Scale, Working Memory Scale, Plan/Organize Scale, Organization of Materials Scale, Monitor Scale). BRS = Behavior Rating Scale, see Bayley. CA = corrected age (corrected for preterm birth, <37 weeks’ gestation). CBCL = Child Behavior Checklist (subscales: internalizing, externalizing; for older children—Competence). CDI = Child Development Inventory (subscale: Social). Conners 3^TM^ AI-P = Conners 3rd Edition ADHD/DSM-IV Index-Parent. Ctrl = control group. d = day(s). Denver = Denver Developmental Screening Test (subscale: Personal–Social). DHA = docosahexaenoic acid. FF = formula fed. GMDS = Griffiths Mental Development Scale (subscale: Personal–Social). GRS = Gifted Rating Scale. IBQ/ECBQ = Infant Behavior Questionnaire-Revised/Early Childhood Behavior Questionnaire (no overall score, IBQ scales: activity level, distress to normal stimuli, distress to limitations, soothability, smiling and laugher, duration of orienting/ECBQ scales: effortful control, activity level). ITQ = Infant Temperament Questionnaire (overall, subscales: fussy-difficult, unadaptable, dull, unpredictable). KPSDSI = Knobloch, Passamanick and Sherrard’s Developmental Screening Inventory (subscales: Adaptive, Personal–Social). mo = month(s). No diff = no difference. NR =n reported. n-3 = omega-3 long-chain polyunsaturated fatty acid. PDDST = Pervasive Developmental Disorders Screening Test. Preg = pregnancy. R = revised. Ref = reference group. RITQ = Revised Infant Temperament Questionnaire (overall, subscales: activity, rhythmicity, approachability, adaptability, intensity, mood, persistence, distractibility, threshold). SDQ = Strengths and Difficulties Questionnaire (Total Difficulties score overall, subscales: emotional symptoms, hyperactivity, conduct problems, peer problems, prosocial behavior, [Impact, optional]). SES = socioeconomic status. STSC = Short Temperament Scale for Children (Overall, subscales: approach, inflexibility, persistence, rhythmicity). TRF = Teacher Report Form. Trt = treatment group. UK = United Kingdom. USA = United States of America. VABS = Vineland Adaptive Behavior Scales. wk = week(s). Wolke = modified Wolke scale (no overall score, scales: activity, emotional tone, responsiveness to examiner, cooperation, vocalization). y = years.
